# A Transcranial Magnetic Stimulation Trigger System for Suppressing Motor-Evoked Potential Fluctuation Using Electroencephalogram Coherence Analysis: Algorithm Development and Validation Study

**DOI:** 10.2196/28902

**Published:** 2021-06-07

**Authors:** Keisuke Sasaki, Yuki Fujishige, Yutaka Kikuchi, Masato Odagaki

**Affiliations:** 1 Department of Environment and Life Engineering Graduate School of Maebashi Institute of Technology Maebashi Japan; 2 Department of Systems Life Engineering Maebashi Institute of Technology Maebashi Japan; 3 Department of Rehabilitation for Intractable Neurological Disorders Institute of Brain and Blood Vessels Mihara Memorial Hospital Isesaki Japan; 4 Division of Systems Life Engineering Graduate School of Maebashi Institute of Technology Maebashi Japan

**Keywords:** motor-evoked potential, transcranial magnetic stimulation, electroencephalogram, coherence, variability, fluctuation, trigger, threshold, coefficient of variation, primary motor cortex

## Abstract

**Background:**

Transcranial magnetic stimulation (TMS), when applied over the primary motor cortex, elicits a motor-evoked potential (MEP) in electromyograms measured from peripheral muscles. MEP amplitude has often been observed to fluctuate trial to trial, even with a constant stimulus. Many factors cause MEP fluctuations in TMS. One of the primary factors is the weak stationarity and instability of cortical activity in the brain, from which we assumed MEP fluctuations originate. We hypothesized that MEP fluctuations are suppressed when TMS is delivered to the primary motor cortex at a time when several electroencephalogram (EEG) channels measured on the scalp are highly similar in the frequency domain.

**Objective:**

We developed a TMS triggering system to suppress MEP fluctuations using EEG coherence analysis, which was performed to detect the EEG signal similarity between the 2 channels in the frequency domain.

**Methods:**

Seven healthy adults participated in the experiment to confirm whether the TMS trigger system works adequately, and the mean amplitude and coefficient of the MEP variation were recorded and compared with the values obtained during the control task. We also determined the experimental time under each condition and verified whether it was within the predicted time.

**Results:**

The coefficient of variation of MEP amplitude decreased in 5 of the 7 participants, and significant differences (*P=.*02) were confirmed in 2 of the participants according to an *F* test. The coefficient of variation of the experimental time required for each stimulus after threshold modification was less than that without threshold modification, and a significant difference (*P*<.001) was confirmed by performing an *F* test.

**Conclusions:**

We found that MEP could be suppressed using the system developed in this study and that the TMS trigger system could also stabilize the experimental time by changing the triggering threshold automatically.

## Introduction

Transcranial magnetic stimulation (TMS) is a noninvasive method of stimulating cortical neurons [1]. The stimulus coil placed on the scalp generates induced electric fields in the brain, which then stimulate cortical neurons. TMS over the primary motor cortex (M1) has been used to evaluate corticospinal excitability in perioperative assessment [2]. When TMS is delivered to the M1, the efferent signal passes through the corticospinal tract [3]; consequently, the motor-evoked potential (MEP) can be measured using an electromyogram (EMG) of the peripheral muscle with a latency of approximately 20 ms following TMS. The amplitude of the MEP in TMS is often unstable and fluctuates even under similar conditions [4-8]. There are several possible factors that affect the variability of MEP amplitude, which vary depending on internal and external factors [9,10]. Furthermore, there are many factors involved, such as changes in body temperature, blood pressure, the atmosphere in the laboratory, and the participant’s posture. It is thus difficult to identify the factors that affect MEP fluctuations. If the fluctuation of the MEP amplitude can be suppressed, this suppression method could be applied in a wide range of fields.

We assumed that one possible factor of MEP fluctuation in TMS was the change in the state of cortical stationarity. Cortical excitability can be measured using an electroencephalogram (EEG) [11]. The similarity of the measured EEG is calculated using coherence analysis, which is a method for calculating the correlation between 2 EEG signals in the frequency domain. We hypothesized that the fluctuation of MEP amplitude must be suppressed when TMS is delivered to the M1 at a time when the electroencephalograms of 2 channels measured on the scalp are highly similar in the frequency domain. In addition, we surmised that the experimental time should be controlled to maintain the accuracy of the experimental data. In this study, we developed an online TMS trigger system for the suppression of MEP fluctuations using EEG coherence analysis while controlling the experimental time.

## Methods

### TMS Trigger System

**Figure 1:**
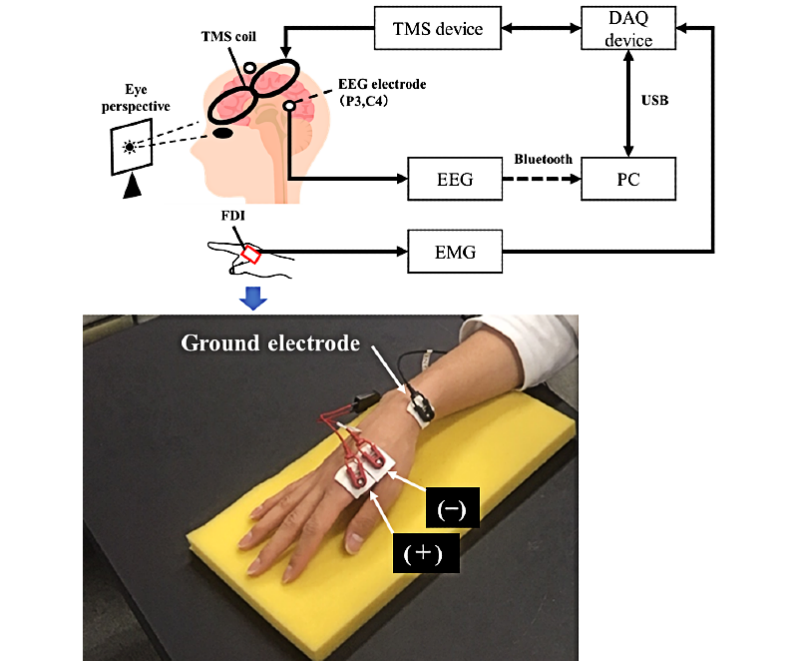
TMS trigger system using EEG coherence analysis. DAQ: data acquisition device; EEG: electroencephalogram; EMG: electromyogram; FDI: first dorsal interosseous; TMS: transcranial magnetic stimulation.

Figure 1 shows an overview of the proposed system. This system is composed of a single-pulse TMS device (Magstim 200^2^, Magstim Co, Ltd), an EEG device (Polymate Mini AP108, Miyuki Giken Co, Ltd,), an IW2PAD EMG (frequency characteristics: 5.3-442 Hz, common-mode rejection ratio: 94 dB; Oisaka Electronic Equipment Ltd), a data acquisition device (USB-6210, National Instruments), and a PC. Software for sending the trigger signal to the TMS device under specific EEG conditions was developed. EEG data measured from P3 and C4 of the international 10–20 system were continuously transmitted to a PC via Bluetooth. The EEG was recorded at a sampling rate of 500 Hz and filtered using a fourth-order Butterworth bandpass filter with a cutoff frequency of 1 Hz to 30 Hz. The EMG results were measured using a data acquisition device connected via a USB. An online EEG analysis was performed during the trial. The triggering signal was sent to the TMS device when the preset TMS condition was satisfied; the data acquisition device then began measuring the MEP waveform from the first dorsal interosseous muscle of the right index finger using the EMG device. The EMG was recorded at a sampling rate of 5 kHz.

**Figure 2:**
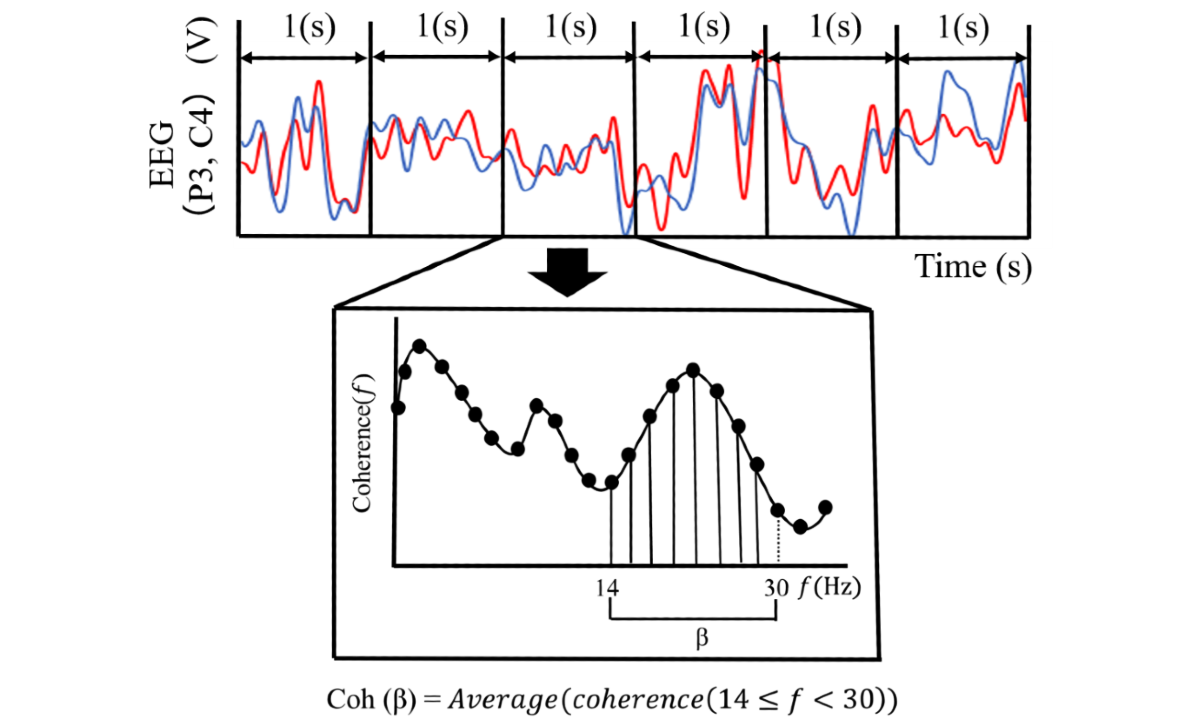
Data processing. The coherence function indicates the frequency spectrum of correlation between 2 EEG channels (P3 and C4). Coh (*β*) is defined as the average value of coherence between 14 Hz and 30 Hz. The values of Coh (*β*) are used to determine the transcranial magnetic stimulation trigger timing. EEG: electroencephalogram.

Coherence analysis was performed to detect the EEG signal similarity between the 2 channels in the frequency domain. The coherence of the 2 EEG signals was calculated using the following equation:




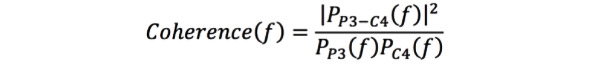




where P_P3_ and P_C4_ are the power spectrum densities of each EEG waveform, and P_P3-C4_ is the cross-power spectrum density of the 2 EEG waveforms. Therefore, the coherence function indicates the similarity between 2 EEG waveforms in the frequency domain. Coh (*β*), which is the area of the coherence function between 14 Hz and 30 Hz, is defined as the average value of coherence in the *β* frequency band (Figure 2).

### TMS Triggering Threshold

**Figure 3:**
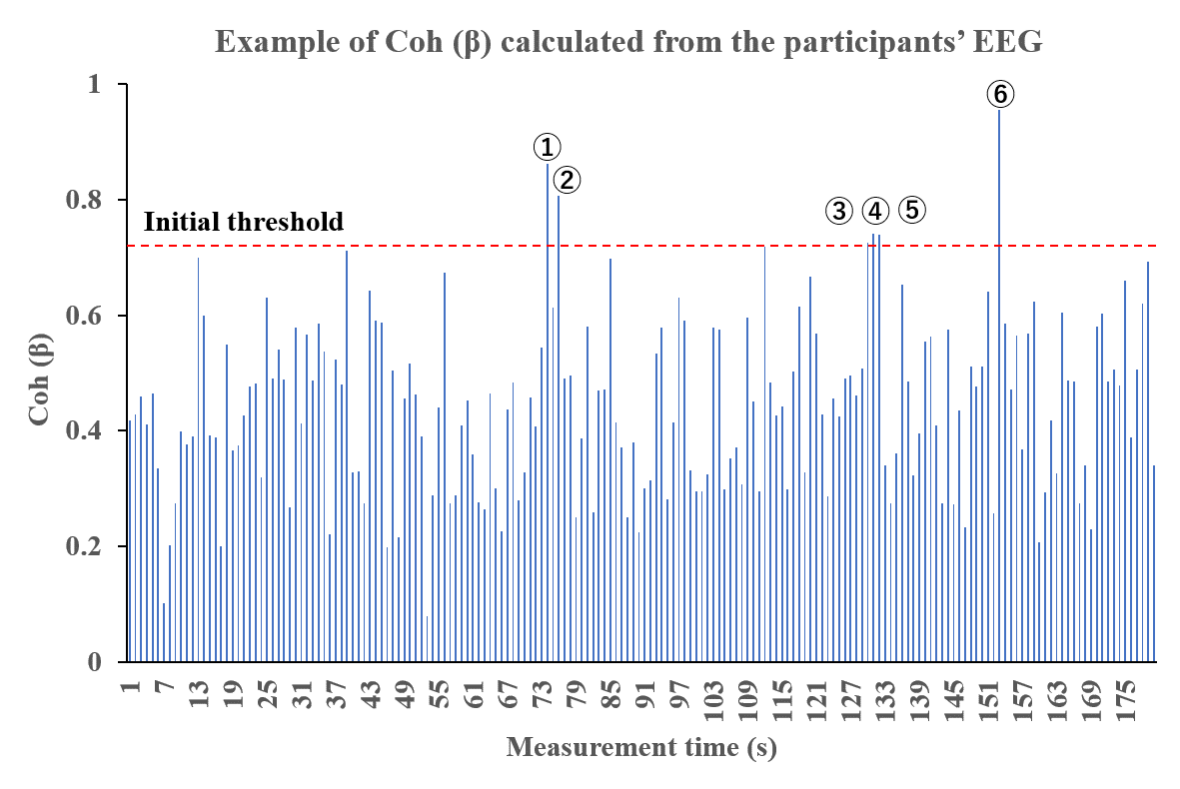
Presetting of the initial threshold. We determined the initial threshold of Coh (*β*) under which the trigger could be performed 6 times in 180 seconds. EEG: electroencephalogram.

Prior to the experiments, we measured the participants’ EEG for 180 seconds, analyzed the 180 data points divided into 1-second fragments, and confirmed the characteristics of Coh (*β*). The system predicted that the trigger could be performed 6 times in 180 seconds under these thresholds; that is, once every 30 seconds on average (Figure 3). Considering that the maximum standby time was 60 seconds in the TMS device, we assumed that TMS should be output in approximately 30 seconds, which was the median of the maximum standby time.

**Figure 4:**
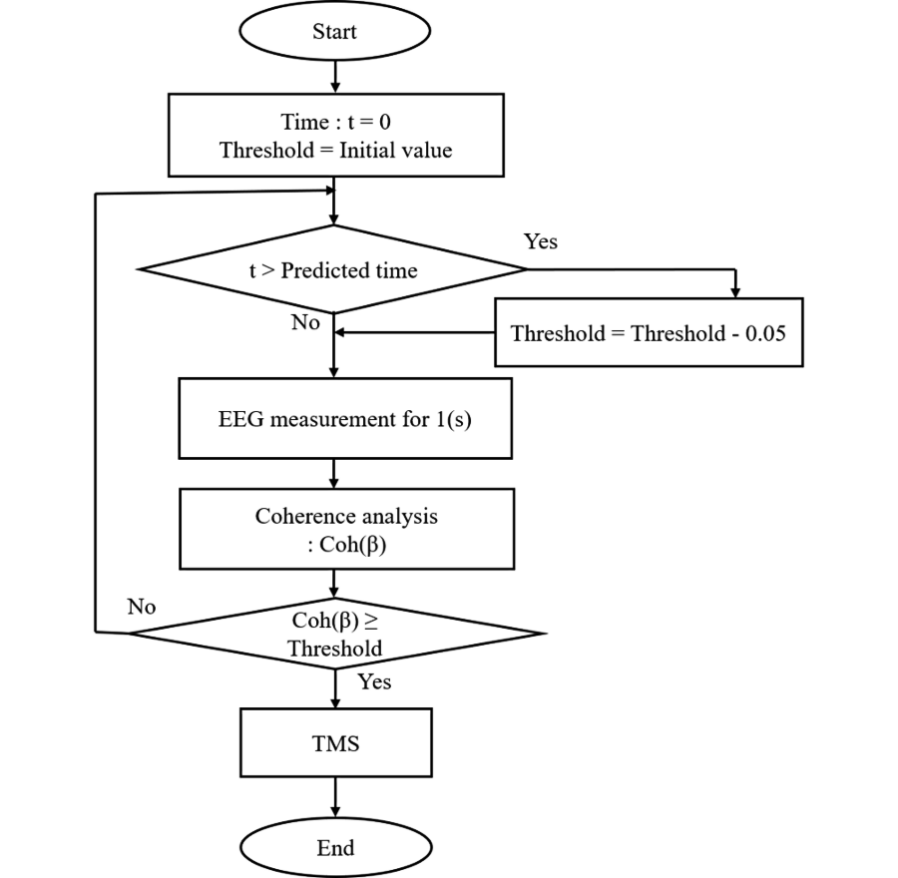
Flowchart of one section of coherence analysis in the TMS trigger system. EEG: electroencephalogram; TMS: transcranial magnetic stimulation.

Figure 4 shows the flowchart of the experimental protocol. In the proposed system, the threshold was automatically modified to be a loose condition if there was no stimulus in 30 seconds. Online coherence analysis was performed on the EEG data once every second during the experiment, and the TMS trigger signal was sent from the data acquisition device to the TMS device at the time when the EEG coherence value of Coh (*β*) was greater than the threshold value (Coh [*β*] ≥ threshold), and TMS was applied to the M1 immediately.

The EEG coherence analysis was paused following TMS and then resumed after 10 seconds. Because the maximum standby time was 60 seconds in the TMS device, the capacitor bank in the TMS device was manually charged if there was no TMS over 60 seconds. If 30 seconds passed from the beginning of the experiment, the threshold of Coh (*β*) was updated with a ∆threshold = –0.05 for every second until the TMS was applied. Once the TMS trigger was output, the threshold value was set to the initial value.

### Experimental Evaluation of the TMS Trigger System

We performed an experimental evaluation of the TMS triggering system to suppress MEP fluctuation and TMS trigger timing. The participants for the experiment included 7 healthy adults (6 males and 1 female; mean age 26 years, SD 8.2 years). None of the participants had a history of physical neuropathy or epilepsy. Prior to the experiments, written informed consent based on the Declaration of Helsinki was obtained from all participants for publication. All procedures used in this study were approved by the ethics committee of the Maebashi Institute of Technology.

The participants were asked to gaze at a single point with their eyes open while at rest, TMS over the left M1 was applied, and MEP caused by TMS was measured from the first dorsal interosseous muscle of the right index finger. We applied TMS 10 times in total, and the stimulation intensity was 150% of the resting motor threshold. The trigger condition was set as Coh (*β*) ≥ threshold, and the MEP was derived using the TMS trigger system. As the control task, TMS was applied at random intervals between 25 and 35 seconds to stimulate M1 and derive MEP without using the TMS trigger system.

To confirm whether the TMS system was effective, the mean amplitude and coefficient of variation of the MEP were recorded and compared with the values in the control task. We also determined the experimental time under each condition and verified whether it was within the predicted time.

## Results

Table 1 summarizes the initial trigger thresholds for all 7 participants in the stimulus trigger condition of Coh (*β*) ≥ threshold. These threshold values were obtained from the EEG data from 180 seconds of testing. In this experiment, we determined the threshold for TMS output in 30 seconds.

**Table 1:** Summary of initial thresholds of Coh (*β*) for all participants.

Participant	Sex (M/F)	Age (years)	Coh (*β*)^a^
Participant 1	M	24	.660
Participant 2	M	25	.770
Participant 3	M	46	.530
Participant 4	F	22	.570
Participant 5	M	22	.695
Participant 6	M	22	.671
Participant 7	M	22	.670

^a^Coh (*β*): the area of the coherence function between 14 Hz and 30 Hz, defined as the average value of coherence in the *β* frequency band.

**Figure 5:**
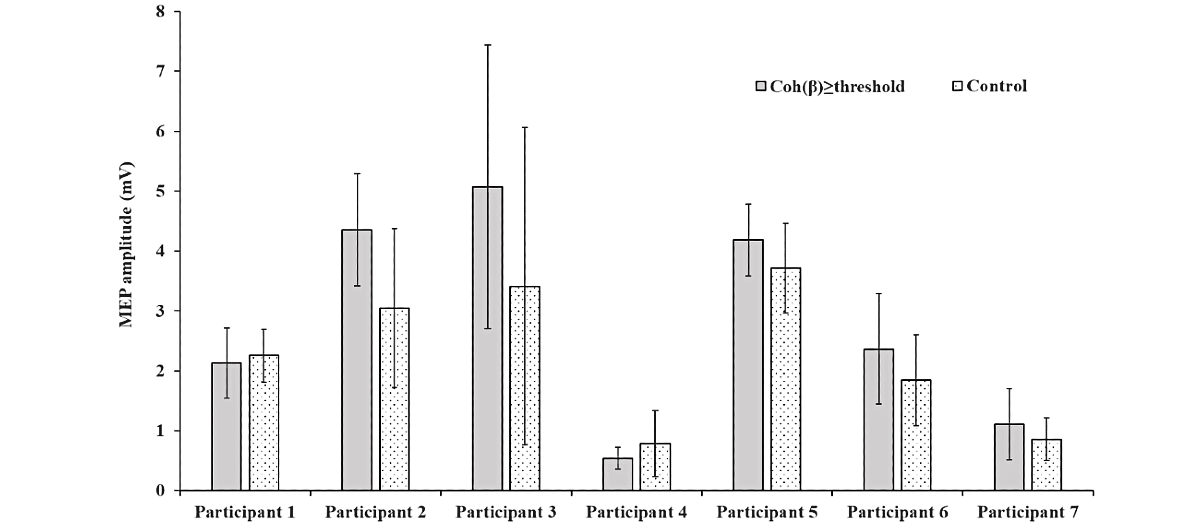
The mean of MEP amplitudes. MEP: motor-evoked potential.

Figure 5 shows the average MEP amplitude measured under trigger conditions and controls for each participant. The vertical axis indicates the average MEP amplitudes in 10 trials, and the error bars represent the SD. As evident in Figure 5, no remarkable changes in the MEPs were observed. Table 2 shows the coefficient of variation (CV) of the MEP amplitude. An *F* test was performed to examine the significant differences. When the trigger condition of Coh (*β*) ≥ threshold was fulfilled, the CV decreased in 5 out of 7 participants, and a significant difference of 5% was confirmed in 2 of the participants.

**Table 2:** Comparison of the coefficients of variation of motor-evoked potential amplitudes (*F* test).

Coefficient of variation value	Participant						
	1	2	3	4	5	6	7
Coh (*β*)^a^ ≥ threshold	0.276	0.215^b^	0.467	0.343^b^	0.143	0.390	0.534
Control	0.196	0.436^b^	0.777	0.699^b^	0.202	0.410	0.419

^a^Coh (*β*): the area of the coherence function between 14 Hz and 30 Hz, defined as the average value of coherence in the *β* frequency band.

^b^A significant difference (*P*=.02) was confirmed in this participant.

Table 3 shows the recorded experimental times. It was confirmed that the actual time without threshold modification was longer than the predicted time in 4 out of 7 participants, but the actual time with threshold modification was longer than the predicted time in 1 out of 7 participants.

Table 4 shows the mean, SD, and CV of the experimental time required for each stimulus in all participants. It was confirmed that the CV of the experimental time required for each stimulus with threshold modification was less than that without threshold modification. An *F* test was performed to determine the significant difference, and a significant difference of 1% was observed.

Table 5 shows the transition of Coh (*β*) at the time of stimulation. The mean value of Coh (*β*) for the 10 stimuli is shown at the bottom.

**Table 3:** Comparison of predicted time and actual elapsed time.

Time		Participant						
		1	2	3	4	5	6	7
Predicted time (seconds)		311	310	312	311	311	312	315
**Actual time (seconds)**								
	Without threshold modification	344^a^	141	174	739^a^	356^a^	453^a^	245
	With threshold modification	251	215	216	223	159	147	354^b^

^a^The actual time without threshold modification was longer than the predicted time.

^b^The actual time with threshold modification was longer than the predicted time.

**Table 4:** Mean, SD, and CV^a^ of experimental time required for each stimulus in all participants (*F* test).

Value	Without threshold modification	With threshold modification
Mean (seconds)	35.0	22.4
SD (seconds)	41.1	13.8
CV	1.17^b^	0.616^b^

^a^CV: coefficient of variation.

^b^A significant difference (*P*<.001) was confirmed.

**Table 5:** Transition of Coh (*β*)^a^ at the time of stimulation. The initial triggering threshold, the transition of Coh (*β*) in 10 stimuli, and the average of Coh (*β*) are shown for each participant.

Value	Participant (Coh [*β*])						
	1	2	3	4	5	6	7
Initial triggering threshold	≥.660	≥.770	≥.530	≥.570	≥.695	≥.671	≥.670
1st time	.698	.856	.624	.635	.720	.672	.672
2nd time	*.557^b^*	.777	.632	*.388*	.937	.676	*.341*
3rd time	*.363*	.792	.569	.580	*.612*	*.748^c^*	*.557*
4th time	.718	.786	*.627^c^*	.950	.754	*.724^c^*	*.271*
5th time	.700	*.828^c^*	.551	.600	.962	.762	*.606*
6th time	.661	.781	.571	*.416*	*.657*	.696	*.403*
7th time	.664	.826	.545	*.445*	.719	.702	*.359*
8th time	.684	*.744*	*.620*	.669	.746	.671	*.633*
9th time	*.435*	.817	.559	.648	.955	.685	*.475*
10th time	*.607*	*.579*	*.430*	.684	*.442*	.672	*.322*
Mean	.609	.773	.560	.601	.750	.692	.464

^a^Coh (*β*): the area of the coherence function between 14 Hz and 30 Hz, defined as the average value of coherence in the *β* frequency band.

^b^Italics indicate cases where the initial triggering threshold was modified to be set lower.

^c^Coh (*β*) at the time of stimulation satisfied the initial triggering threshold even when the threshold was modified to be set lower.

## Discussion

### Principal Findings

The number of cases where the initial triggering threshold was modified to be set lower were 3 or fewer in 10 stimulus incidences in all participants except for participants 1 and 7 (Table 2). We further found that the CV decreased in all participants except for participants 1 and 7 (Table 2). These results indicate that the fluctuation of MEP amplitude decreased when the modification of the initial triggering threshold of TMS did not occur frequently. In addition, the mean values of Coh (*β*) from all participants except for participants 1 and 7 (Table 5) exceeded the initial triggering threshold. This means that the proposed system worked effectively, and the initial triggering threshold was appropriate in these cases. Although the initial triggering threshold was modified in participants 2, 3, and 6 (Table 5), the Coh (*β*) at the time of stimulation eventually exceeded the initial triggering threshold. Thus, it is probable that the change in EEG was transient.

The triggering threshold was modified 4 and 9 times out of the 10 stimuli in participants 1 and 7 (Table 5), respectively. Because the number of modifications in participants 1 and 7 was larger than that of the others, the actual time with threshold modification for these participants was longer than that of the others. In participant 7, it was confirmed that the actual time was longer than the predicted time. The MEP fluctuations in these 2 participants were not suppressed because the mean values of Coh (*β*) at the stimulation in participants 1 and 7 were smaller than the initial thresholds. This fact paradoxically suggests that MEP fluctuations are suppressed when Coh (*β*) is high. In participant 7, the system modified the initial threshold in 9 of the 10 stimulus incidences, so it is probable that the participant was in an unsteady state at the time of the experiment.

### Comparison With Previous Studies

Ogata et al [12] suggested that M1 excitability can be predicted by EEG oscillations before TMS. The MEP amplitude increased when the *α* band’s power was high, and the power of the *β* band did not affect the MEP amplitude. In addition, several studies have shown that β oscillations are inhibited when MEP amplitudes increase [12-16]. Generally, the *α* wave decreases and the *β* wave increases when the participants do not close their eyes. Considering that the coherence analysis used in this study quantified the similarity of EEG power and the MEP from participants who did not close their eyes, it is probable that the MEP fluctuation was suppressed because the *β* band’s power level was high.

### Interpretation of the Findings

It is suggested that the system we developed could suppress the fluctuation of MEP amplitude under the steady state and could also reduce the variation in the experimental time required for each stimulus, as shown in Table 4. We concluded that avoiding an unexpected extension of the experimental time can stabilize the participant’s condition and contribute to improving the accuracy of the experimental data.

### Strengths and Limitations

The system developed in this study had several limitations. It was difficult to determine the steady state of the participant and the timing at which the MEP amplitude could be efficiently suppressed. Owing to the specifications of the TMS device, we set the initial triggering threshold at the time when it appeared, approximately once every 30 seconds. There is no guarantee that this initial value is determined while the participant is in the steady state. If the initial value is determined while the participant is in the unsteady state, the MEP amplitude may not be adequately suppressed. If the triggering threshold is higher, the MEP amplitude may be suppressed more, but considering the burden on the participant and the accuracy of the experimental data, we prefer to shorten the experimental time. Therefore, we developed a system that modifies the triggering threshold. In this system, if the initial triggering threshold is not satisfied, it is lowered to avoid an extended experimental time. However, if the participant remains in an unsteady state, the triggering threshold may remain low, and eventually, the MEP amplitude may not be suppressed. Therefore, it is difficult to determine how high the initial triggering threshold, Coh (*β*), should be, and further verification is required.

No other studies that suppressed MEP amplitude fluctuations using EEG coherence analysis exist besides this study. The EEG rhythm comprehensively reflects the biological response to external and physiological factors [17-19]. This system has the advantage that the state of the brain can be stabilized by monitoring the EEG similarity between the left and right hemispheres when TMS is delivered to the M1. In addition, this system enables control of the actual experimental time as well as the suppression of MEP amplitude fluctuations. We are certain that this is an essential element for practical clinical use.

### Future Perspectives

In clinical practice, MEP is measured to avoid nerve damage during neurosurgery and orthopedic surgery [20]. Even under similar conditions, the MEP amplitude often fluctuates, and it is difficult to identify the cause immediately [4,5]. If the fluctuation of MEP amplitude that is unrelated to the surgical operation can be suppressed, the incidence of false positives can be reduced, which can contribute to patient safety. During the operation, both rapid procedures and the suppression of MEP amplitude fluctuation are required to avoid placing a burden on the patient.

The current system can only set the triggering threshold lower when the actual time is longer than the predicted time. If the system can set the triggering threshold higher or lower in response to changes in the participant’s condition, the actual experimental time will be closer to the predicted time and MEP fluctuation will be significantly suppressed. In addition, if the system can be improved in future studies and determine whether a participant is in a steady state online while measuring the MEP, the triggering threshold can be changed more responsively, and the fluctuation of MEP amplitude that causes false positives can be suppressed. It can also help avoid perioperative complications. Thus, we will continue to improve our system to contribute to clinical applications.

### Conclusions

We developed a TMS trigger system to suppress MEP fluctuations using feedback-type EEG coherence analysis. We suggest that the fluctuations in MEP amplitude could be suppressed by applying TMS to the M1 when Coh (*β*) is high while controlling the experimental time.

## Abbreviations

CV: coefficient of variation
EEG: electroencephalogram
EMG: electromyogram
MEP: motor-evoked potential
M1: primary motor cortex
TMS: transcranial magnetic stimulation
